# Please inhibit responsibly: Natural and synthetic actin toxins as useful tools in cell biology

**DOI:** 10.1091/mbc.E25-05-0264

**Published:** 2025-08-13

**Authors:** Katrina B. Velle, Masayuki Onishi

**Affiliations:** ^a^Department of Biology, University of Massachusetts Dartmouth, North Dartmouth, MA 02747; ^b^Department of Biology, Duke University, Durham, NC 27708; Seattle Children's Research Institute & University of Washington

## Abstract

The actin cytoskeleton drives many critical cell functions, including motility, division, and vesicular trafficking. To fulfill these functions, actin networks are dynamic and tightly regulated by dozens of proteins that cause actin to assemble and disassemble at the proper time and place. Given the importance of actin to a cell's biology, it is not surprising that some organisms produce toxins that target actin dynamics to incapacitate prey, win turf wars, or as a defense against predation. For decades, cell biologists have leveraged these toxins and synthesized new ones to cause defects in the structure and function of the actin cytoskeleton. Here, we provide an overview of commonly used actin inhibitors and their origins, as well as best practices for their use in biological studies.

## INTRODUCTION

The actin cytoskeleton is a network of actin filaments and actin-binding proteins that support and remodel membranes to control many cellular processes. Precisely regulating the assembly of actin monomers into polymers and higher-order structures, as well as controlling their disassembly, allows actin networks to be responsive and dynamic (Velle *et al.*, 2024). Small molecules that bind either actin monomers or polymers to prevent these dynamics, as well as those that bind to actin-binding proteins and indirectly alter actin dynamics or function, can therefore cause actin-dependent phenotypes. In this Perspective, we provide a brief overview of such “actin inhibitors” that have potential applications in organisms across the eukaryotic tree. We discuss their mechanisms of action, origins, and some cautions in using them in cell biological experiments, especially in nontraditional model systems.

## INHIBITORS OF ACTIN MONOMERS AND POLYMERS

**FIGURE 1:**
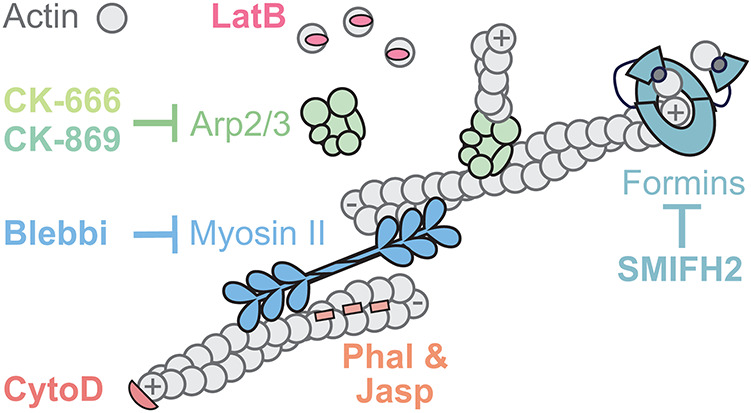
Actin inhibitors target many aspects of actin dynamics and/or organization. Commonly used actin drugs are shown as a cartoon with inhibitors that bind actin shown at their approximate binding sites, and inhibitors of actin-binding proteins shown using inhibitory arrows.

Some actin inhibitors bind directly to actin monomers (Figure 1). For example, **latrunculins** (LatA and LatB) bind to the ATP/ADP-binding cleft of actin monomers at 1:1 stoichiometry (Morton *et al.*, 2000), which is thought to sequester actin from polymerization. Because many actin networks undergo fast turnover, preventing newly disassembled actin monomers from repolymerizing likely results in the observed rapid loss of actin structures within the cell (Spector *et al.*, 1983; Ayscough *et al.*, 1997). Their rapid effects, potency, membrane permeability, and reversibility have made latrunculins some of the most widely used “go-to” actin inhibitors in cell biological research. Like latrunculins, **swinholide A** is an inhibitor that results in the net loss of actin polymer by binding to actin monomers, although at 1:2 stoichiometry (Bubb *et al.*, 1995), suggesting that this drug binds and sequesters actin dimers. In addition to inhibiting new actin polymerization, both swinholide A and LatA can disassemble existing polymers by severing (Bubb *et al.*, 1995; Fujiwara *et al.*, 2018), and LatA can also promote disassembly at filament ends (Fujiwara *et al.*, 2018).

Many other classes of inhibitors bind to different surfaces of actin filaments (Figure 1). **Cytochalasins** (such as cytochalasins B and D), for example, cap the rapidly growing “plus” (also called “barbed”) end of an actin filament, thereby blocking the incorporation of additional monomers and causing a net loss of actin filaments (MacLean-Fletcher and Pollard, 1980; Cooper, 1987). Due to their efficacy and early discovery in 1960s, cytochalasins B and D were used in foundational studies elucidating the role of actin filaments in various cellular processes, such as cytokinesis, morphogenesis, and muscle contraction in animals, and chloroplast movement in green algae (Carter, 1967; Schroeder, 1970; Wessells *et al.*, 1971; Wagner *et al.*, 1972). Other inhibitors, like **jasplakinolide** and **phalloidin**, bind along the length of actin filaments, stabilizing them by interacting with three subunits (Cooper, 1987; Bubb *et al.*, 1994; Pospich *et al.*, 2020). Jasplakinolide is membrane permeable, and when used in vivo, it hyperstabilizes preexisting actin filaments and induces polymerization (Bubb *et al.*, 1994). Fluorescently conjugated derivatives of jasplakinolide, like SiR-actin, have also been useful in low concentrations for live-imaging studies (Lukinavicius *et al.*, 2014) (also see Melak *et al.*, 2017 for additional live imaging probes). In contrast, phalloidin is membrane impermeable, limiting its uses for live cells except for some special cases (e.g., microinjection; [Hamaguchi and Mabuchi, 1982; Schmit and Lambert, 1990; Wehland *et al.*, 1977]). For experiments using fixed cells, however, fluorescently conjugated phalloidin is used widely and considered the gold standard, to which other actin-visualization methods are often compared (Wulf *et al.*, 1979; Melak *et al.*, 2017).

## INHIBITORS OF ACTIN-BINDING PROTEINS

In contrast with small molecules that bind actin directly to influence its dynamics, other compounds target actin-binding proteins (Figure 1). Actin rarely assembles spontaneously in cells, and instead is highly regulated by layers of upstream proteins and signals (Velle *et al.*, 2024). Among these regulators are actin nucleators—a class of proteins and complexes that initiate the assembly of a new filament by stabilizing an actin dimer or trimer. Inhibitors of actin nucleators and other actin-binding proteins can therefore be used to impair specific types of actin networks and activities.

Branched actin networks are assembled by the Arp2/3 complex—an actin nucleator that, when activated, can bind to the side of a preexisting filament and initiate the assembly of a new filament to form a branch (Mullins *et al.*, 1998). These networks are ideal for generating expansive forces that push on membranes. Arp2/3-derived actin networks drive myriad cellular processes, including crawling motility, vesicular trafficking, endocytosis, and phagocytosis (Velle *et al.*, 2024), making the Arp2/3 complex a useful target for studying these phenotypes. The small-molecule **CK-666** directly binds the Arp2/3 complex at the interface of Arp2 and Arp3 subunits, and prevents it from nucleating new filaments (Baggett *et al.*, 2012; Hetrick *et al.*, 2013; Nolen *et al.*, 2009). Similarly, the small-molecule **CK-869** locks the Arp2/3 complex in an inactive conformation by binding to Arp3 (Hetrick *et al.*, 2013; Nolen *et al.*, 2009). These compounds have been useful for studies of diverse systems, including plant cells (Xu *et al.*, 2024), single-celled eukaryotes (Velle and Fritz-Laylin, 2020; Prostak *et al.*, 2021; Bigge *et al.*, 2023a), animal cells (e.g., lamellipodia in sea urchin cells [Henson *et al.*, 2015] and mammalian cells [Abu Taha *et al.*, 2014; Haynes *et al.*, 2015], as well as  spindle positioning and cytokinesis in mouse oocytes [Sun *et al.*, 2011; Yi *et al.*, 2011]), and for investigating host–pathogen interactions (Dhanda *et al.*, 2018; Miller *et al.*, 2018; Pfanzelter *et al.*, 2018; Velle and Campellone, 2018; Malych *et al.*, 2025). Looking upstream of the Arp2/3 complex, **wiskostatin** is a compound that binds the Arp2/3 complex activator N-WASP, locking it in its autoinhibited conformation (Peterson *et al.*, 2004). Although useful for impairing *only* the N-WASP pathway of Arp2/3 activation (and not the ∼10 other activators), wiskostatin has well-documented off-target effects such as reducing cellular ATP levels (Guerriero and Weisz, 2007).

Contractile actin networks often drive phenotypes like migration, cytokinesis, and membrane blebbing (Velle *et al.*, 2024). These networks typically involve actin filaments assembled by formin family proteins and require the motor protein myosin II. Formins can both nucleate and elongate actin filaments, and these activities can be perturbed by **SMIFH2**, a broad inhibitor of formin family proteins thought to bind the FH2 domain (Rizvi *et al.*, 2009). Additionally, SMIFH2 has been shown to inhibit multiple myosins (Nishimura *et al.*, 2021), which makes this drug potentially useful for broadly assessing the roles of actin and myosin networks. However, due to this lack of specificity (likely due to high electrophilicity [Baell, 2010]) and additional known off-target effects (Innocenti, 2023), caution should be taken in interpreting any result with SMIFH2.

Myosin II can also be specifically inhibited by the small-molecule **blebbistatin** (Straight *et al.*, 2003). Blebbistatin works by binding myosin II when its motor head is complexed with ADP-Pi and slowing the release of phosphate (Kovacs *et al.*, 2004). Combining blebbistatin with fluorescence imaging can be problematic, as it is inactivated by blue light (Kolega, 2004; Sakamoto *et al.*, 2005), making it incompatible with GFP-tagged proteins and some fluorescent dyes. Although this could be leveraged as a way to inactivate blebbistatin in specific cells or subcellular regions, caution should be taken as it becomes toxic in cells following blue light inactivation (Kolega, 2004; Sakamoto *et al.*, 2005; Mikulich *et al.*, 2012). Due to these problems and its low solubility in aqueous solutions, several derivatives of blebbistatin like para-aminoblebbistatin have become popular alternatives (Kepiro *et al.*, 2012; Kepiro *et al.*, 2014; Varkuti *et al.*, 2016). Blebbistatin and its derivatives have been used for studying myosin II-dependent processes in budding and fission yeasts (Mishra *et al.*, 2013; Malla *et al.*, 2022), chytrid fungi (Robinson *et al.*, 2022), *Dictyostelium* amoebae (Shu *et al.*, 2005), and mammalian cells (Guha *et al.*, 2005; Murthy and Wadsworth, 2005). Although these examples represent a wide array of diverse organisms, blebbistatin is notably ineffective against *Drosophila melanogaster's* myosin II in cells and in vitro (Straight *et al.*, 2003; Heissler *et al.*, 2015), highlighting the importance of carefully testing even widely used drugs when introducing them into a new system.

## ORIGINS AND ECOLOGICAL ROLES OF ACTIN INHIBITORS

**FIGURE 2:**
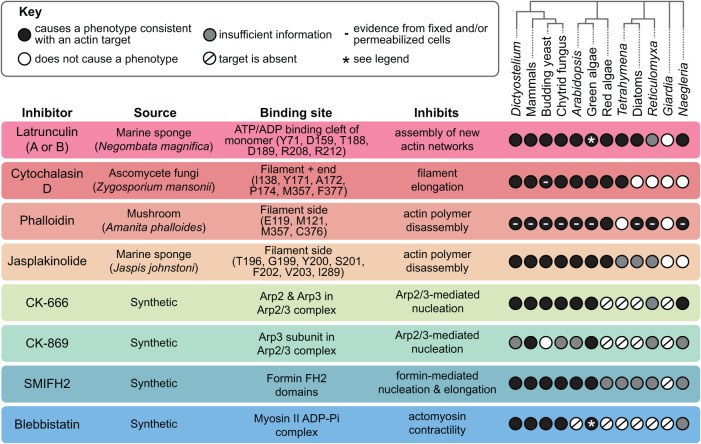
Frequently used inhibitors can impair actin dynamics in many but not all species. Inhibitors are listed in a table with their source, binding site, and target. For actin-binding inhibitors, a few key residues for binding corresponding to the full rabbit muscle actin sequence (XP_002722940.2) are listed, although this list is not exhaustive (see also: Supplemental Figures S5–S6 in [Velle and Fritz-Laylin, 2020], Figure 1 in [Wirshing *et al.*, 2025] and [Faulstich *et al.*, 1993; Belmont *et al.*, 1999; Morton *et al.*, 2000; Nair *et al.*, 2008; Pospich *et al.*, 2017; Kumari *et al.*, 2020]). Model organisms and their phylogenetic relationships are also shown, with circles, indicating if an inhibitor causes an actin-related phenotype (black circle), if it has been tested but does not cause a phenotype (white circle), if the target proteins are absent (slash), or if there is insufficient information to determine if an inhibitor is effective against actin networks in that organism (gray circle). The effects of phalloidin are based on positive actin staining using fixed and/or permeabilized cells, except for a few microinjection experiments and experiments using red algae. Cytochalasin D has only been shown to be effective in permeabilized yeast. Asterisks: LatB is effective against one actin isoform (encoded by IDA5 gene) but not another (NAP1) in *Chlamydomonas*; *Chlamydomonas* lacks Myosin II, but blebbistatin has effects that may be consistent with inhibiting other myosins. **Figure References:** *Dictyostelium*: (Gerisch *et al.*, 2004; Shu *et al.*, 2005; Mondal *et al.*, 2008; Yumura *et al.*, 2014; Dong *et al.*, 2016; Ecke *et al.*, 2020); Mammals (mouse, rat, rabbit, or human): (Carter, 1967; Spector *et al.*, 1983; Bubb *et al.*, 2000; Straight *et al.*, 2003; Nolen *et al.*, 2009; Rizvi *et al.*, 2009); Budding yeast (*Saccharomyces cerevisiae*): (Adams and Pringle, 1984; Li *et al.*, 1995; Ayscough *et al.*, 1997; Nolen *et al.*, 2009; Mendes Pinto *et al.*, 2012; Mishra *et al.*, 2013; Toshima *et al.*, 2016); Chytrid fungus (*Batrachochytrium dendrobatidis*): (Fritz-Laylin *et al.*, 2017; Prostak *et al.*, 2021; Robinson *et al.*, 2022); *Arabidopsis*: (Sawitzky *et al.*, 1999; Collings *et al.*, 2006; Cao *et al.*, 2016; Xu *et al.*, 2024); Green Algae (*Chlamydomonas* or *Micrasterias*): (Hoffman and Goodenough, 1980; Dentler and Adams, 1992; Holzinger and Meindl, 1997; Avasthi *et al.*, 2014; Onishi *et al.*, 2016; Craig *et al.*, 2019); Red Algae (*Porphyra*, *Bostrychia, Cyanidium, or Cyanidioschyzon*): (Suzuki *et al.*, 1995; Wilson *et al.*, 2002; Ackland *et al.*, 2007; Maschmann *et al.*, 2020); *Tetrahymena*: (Hirono *et al.*, 1989; Zackroff and Hufnagel, 2002; Hosein *et al.*, 2005); Diatoms (various species): (Poulsen *et al.*, 1999); *Reticulomyxa*: (Koonce *et al.*, 1986); *Giardia*: (Paredez *et al.*, 2011); *Naegleria*: (Velle and Fritz-Laylin, 2020).

Although CK-666, CK-869, SMIFH2, and blebbistatin are all synthetic compounds, many inhibitors of actin networks are derived from nature (Figure 2). Actin toxins are produced by a diverse array of organisms, including marine sponges (latrunculins [Kashman *et al.*, 1980] and swinholide A [Carmely and Kashman, 1985]), fungi (cytochalasins [Aldridge *et al.*, 1967] and phalloidin [Wieland, 1968]), dinoflagellates (pectenotoxin [Yasumoto *et al.*, 1984] and goniodomin [Sharma *et al.*, 1968]), and bacteria (occidiofungin [Lu *et al.*, 2009] and ACD toxins [Sheahan *et al.*, 2004]). Some of these toxins may prevent predation; pectenotoxin, for example, is produced by dinoflagellates and has been suggested as a defense against copepod grazing (Pourdanandeh *et al.*, 2025). Some others may be used in turf wars for resources between species; cytochalasins and other fungicides are produced at the site of contact when two fungi are cocultured (Knowles *et al.*, 2019). Toxins may even function as offensive weapons, as some cytochalasins confer the ability of fungal pathogens to infect plants (Saiwai *et al.*, 1983; Tsurushima *et al.*, 2005). Compared with our understanding of how these compounds work in some model cells in the laboratory and in experiments in vitro, little is known about how they are synthesized, their natural targets, or how the targeted cells respond to them. Here, we detail one of the most well-studied actin inhibitors, latrunculin.

Latrunculins are isolated from the Red Sea sponge *Negombata magnifica* (formerly *Latrunculia magnifica*) (Nèeman *et al.*, 1975; Spector *et al.*, 1983). Although LatA can be synthesized in vitro (Furstner *et al.*, 2007), the biosynthesis pathway in the sea sponge is still unknown and may involve the sponge's symbionts. Once produced, sponges may use the toxin for self-defense against predators such as carnivorous fish (Nèeman *et al.*, 1975; Proksch, 1994). Alternatively, these toxins may be utilized to “paralyze” the prey microbes that sponges filter-feed upon. Regardless of what latrunculins are used for, a question is raised: How are sponges not intoxicated by their own latrunculins? One potential answer is by diversifying their actin sequences. Although genomic information is not available for *N. magnifica*, many sponges encode multiple actin genes. For example, a well-studied sponge, *Halichondria panicea*, has one conventional (accession XP_064390159.1, 97% identical to human α-actin) and one highly divergent actin (accession XP_064405707.1, 67% identical to human α-actin). Because the latter has a substitution in one of the residues involved in direct hydrogen bonding between human α-actin and LatA (tyrosine 71, which is positioned inside the ATP/ADP-binding pocket and substituted with asparagine in XP_064405707 [Morton *et al.*, 2000]), it is possible that this actin does not bind to LatA and therefore confers resistance to the cells expressing this toxin.

This strategy of diversifying actin sequences to achieve resistance is not limited to toxin producers. Nudibranch mollusks in the genus *Chromodoris* feed on sponges and collect LatA, which in turn is used for self-defense against predators (Cheney *et al.*, 2016). Although the precise mechanism of how they avoid self-toxication by LatA is unclear, the divergent actins in their genomes have substitutions in residues involved in LatA-actin binding (Hertzer *et al.*, 2023). In another reported example, the green alga *Chlamydomonas reinhardtii* has two actins, one conventional (IDA5) and one divergent (NAP1) (Kato-Minoura *et al.*, 1997). Interestingly, NAP1 is not expressed in vegetative cells under normal growth conditions, but it is transcriptionally upregulated upon exposure to latrunculins and depolymerization of filaments made of IDA5 (Onishi *et al.*, 2016; Onishi *et al.*, 2018). NAP1 has a substitution at the position equivalent to tyrosine 71 in human α-actin (substituted with histidine), and indeed, NAP1 forms filaments resistant to the toxins. Thus, isolation of *nap1* mutants (which are sensitive to latrunculins) was crucial for studying the roles of actin filaments in processes such as cell division, endocytosis, ciliary assembly, and chloroplast division (Onishi *et al.*, 2016; Onishi *et al.*, 2020; Bigge *et al.*, 2023b; Clark-Cotton *et al.*, 2025). The exact reason why *Chlamydomonas* (and related algae in the Volvocine group; Kato-Minoura *et al.*, 2003) has evolved this apparent defense mechanism against latrunculin toxins is unclear, but divergent actins are found throughout the eukaryotic tree. Some of these actins are reported to be resistant to latrunculins and/or have mutations similar to those reported to inhibit latrunculin-binding (Ayscough *et al.*, 1997; Belmont and Drubin, 1998; Belmont *et al.*, 1999; Morton *et al.*, 2000; Fujita *et al.*, 2003; Procaccio *et al.*, 2006; Schwarzerova *et al.*, 2010; Riviere *et al.*, 2012; Johnston *et al.*, 2013; Onishi *et al.*, 2016; Filipuzzi *et al.*, 2017; Hertzer *et al.*, 2023). Examples include plant *Arabidopsis thaliana* (ACT9, AT2G42090.1), ciliate *Paramecium tetraurelia* (e.g., XP_001459509.1; [Sehring *et al.*, 2007b]), intestinal parasite *Giardia lamblia* (Paredez *et al.*, 2011), tunicate *Ciona intestinalis* (XP_002128924.1), amoeba *Dictyostelium discoideum* (e.g., XP_636187), *Pipistrellus* bats (CAK6444077.1, XP_036265340.1), *Fusarium* fungi (e.g., UPK93335), and *Symbiodinium* dinoflagellates (e.g., CAE7723233.1, CAE7265032.1, CAE7619015.1). These divergent sequences suggest that actins can evolve at some decent rate while maintaining their essential functions, and some of the evolution may occur as a result of molecular cat-and-mouse between actin and actin-targeting toxins. Thus, when using actin inhibitors in experiments, it is important to consider the possibility that the target organism or cell type may exhibit some unexpected resistance or sensitivity to these drugs. Comparisons of in vivo and in vitro results should be done with some caution, especially when actin proteins from different genomes are used in them. Conversely, when we consider the evolution of actin proteins, nature has already done some genetics experiments and provides rich resources that have not been fully utilized (Velle *et al.*, 2024).

## USE AND MISUSE OF ACTIN INHIBITORS

Small-molecule inhibitors have been a staple in actin cytoskeletal research for decades, and continue to be a valuable tool for probing actin-based phenotypes. Many of these inhibitors work on a wide array of model systems and have well-documented activities (Figure 2). Inhibitors also have some advantages over genetic tools: effects typically occur on a rapid timescale—seconds to minutes—and many inhibitors are reversible and can be washed out. Furthermore, because the actin cytoskeleton is vital to so many cellular processes, knocking out actin itself is often not a viable option. Moreover, the formin inhibitor SMIFH2 has an advantage over genetic tools, in that it inhibits many different formins (as well as myosin II) simultaneously, so it can be used to quickly assess the contributions of formin-derived contractile networks to a particular phenotype (this would, of course, be best paired with further experiments, including genetic manipulations of individual genes).

Despite the many advantages of small-molecule inhibitors, certain precautions should be taken, particularly when introducing them to a new model system. Here are some tips:

1. *Check the binding site*: If a particular drug has not been used in the literature for your model system, it is typically worth checking if the residues at the drug's binding site are conserved (Figure 2) (Sehring *et al.*, 2007a; Paredez *et al.*, 2011; Onishi *et al.*, 2016; Velle and Fritz-Laylin, 2020; Wirshing *et al.*, 2025). For example, many ascomycete fungi have a mutation (valine at position 75) in their actin genes at a phalloidin-binding site, and fluorescent phalloidins fail to stain actin filaments in these species. Amazingly, a single “reversion” mutation at this position to isoleucine is sufficient to allow actin visualization in one of the species, *Aureobasidium pullulans* (Wirshing *et al.*, 2025). However, just because a binding site is conserved, or even if a drug binds in vitro, this does not guarantee the compound will be effective in living cells.
2. *Test a wide array of concentrations*: Using the literature as a guide, test a range of concentrations. Effective in vivo drug doses for different organisms and cell types can vary by orders of magnitude. For example, to completely depolymerize actin filaments, 100 µM LatA is needed for budding yeast (Ayscough *et al.*, 1997), while 1 µM is sufficient for mammalian fibroblasts (Spector *et al.*, 1989). Organisms with cell walls, low membrane permeability, and/or strong efflux pumps may require a higher dose of an inhibitor. Alternatively, mutations in efflux pumps (Asadi *et al.*, 2017; Ayscough, 2000), or a pairing with an efflux pump inhibitor such as verapamil (Lukinavicius *et al.*, 2014) can reduce the required concentration. It is best practice to use the lowest concentration that still results in a robust phenotype. A drug that only has an effect at concentrations orders of magnitude above what is needed for a similar organism can be a red flag. Be aware, however, that the efficacy of inhibitors may be variable from one batch/source to another. As an example, LatB (which is produced by a single supplier) efficacy has not been consistent across several lot numbers (e.g., Onishi *et al.*, 2018), which might have contributed to the notion that LatB is less effective/stable than LatA.
3. *Use controls*: Always use a vehicle control and a control with nothing added. These negative controls can be especially important for drugs dissolved in DMSO—a common solvent that can affect a cell's biology (Fukui and Katsumaru, 1980; Holthaus *et al.*, 2018; Verheijen *et al.*, 2019; Prostak *et al.*, 2021). Inactive controls, including CK-689 for CK-666 (Nolen *et al.*, 2009) and the inactive (+)-blebbistatin enantiomer (Straight *et al.*, 2003), may also be useful. Positive controls are helpful if they are available for your system; for example, if knocking down Arp2/3 complex phenocopies CK-666 treatment, and if there are no additive effects when the two are combined, the drug is likely hitting its target (Rotty *et al.*, 2017; Xu *et al.*, 2024).
4. *Beware of off-target effects*: Some inhibitors, including SMIFH2, wiskostatin, and cytochalasins, have well-documented off-target effects (Zigmond and Hirsch, 1972; Jarett and Smith, 1979; Guerriero and Weisz, 2007; Cossar *et al.*, 2023; Innocenti, 2023). If your system is genetically tractable, check inhibitors of actin-binding proteins by knocking out the target of the inhibitor before treatment to reveal other phenotypes caused by the inhibitor (e.g., Shu *et al.*, 2005). If latrunculin is effective in your system, treating with latrunculin first and then adding the inhibitor of interest can show whether a phenotype is actin linked—if the phenotype persists in the absence of actin filaments, the inhibitor is likely off-target. Finally, actin inhibitors can compete with actin-binding proteins, upsetting the normal balance between actin and its regulators (e.g., Klenchin *et al.*, 2003; Wang *et al.*, 2019), and potentially causing emergent phenotypes that are not directly related to the inhibitor's target.
5. *Be mindful of different isoforms*: An inhibitor may not inhibit every version of its target, especially if multiple genes encode multiple versions. In addition to the well-documented latrunculin-resistant actins (see above), in mammalian cells there are eight different possible Arp2/3 complexes, which are differentially inhibited by CK-666 and CK-869 (Cao *et al.*, 2024).

## FUTURE OUTLOOK

Although inhibitors of actin and actin-binding proteins have been used for decades, their relevance is only growing. Testing well-described compounds in new and emerging model systems is important for understanding the fundamentals of their cytoskeletal biology, as well as evolution. For example, recent studies have shown that some Asgard archaeans possess components of a eukaryotic-like cytoskeleton (reviewed in Charles-Orszag *et al.*, 2024). Having inhibitors that can impair these archaeal actin networks could be critical for defining the contributions of actin to their biology and tracing the evolution of actin phenotypes. Additionally, investigating the roles of actin toxins as biological weapons can tell us more about ecology and the context in which some actins may have evolved. Finally, screens of natural and synthetic products have led to the discovery of additional compounds, which could be useful for treating some eukaryotic pathogens like fungal infections (Ravichandran *et al.*, 2019), toxoplasmosis (Kelsen *et al.*, 2023), or malaria (Moussaoui *et al.*, 2023). New screens or modifications of existing compounds could also be useful for identifying new ways to visualize actin in live cells; although SiR actin (based on Jasplakinolide) is a valuable tool for mammalian cells, it has not been successfully used in most other eukaryotic cell types. By continuing to screen both new compounds and new species, actin inhibitors are certain to remain powerful tools for studying cell biology, evolution, ecology, and pathogenesis.

## References

[B1] Abu Taha A, Taha M, Seebach J, Schnittler HJ (2014). ARP2/3-mediated junction-associated lamellipodia control VE-cadherin-based cell junction dynamics and maintain monolayer integrity. Mol Biol Cell 25, 245–256. 24227887 10.1091/mbc.E13-07-0404 PMC3890345

[B2] Ackland JC, West JA, Pickett-Heaps J (2007). Actin and myosin regulate pseudopodia *Ofporphyra Pulchella*(Rhodophyta) archeospores1. J Phycol 43, 129–138.

[B3] Adams AE, Pringle JR (1984). Relationship of actin and tubulin distribution to bud growth in wild-type and morphogenetic-mutant Saccharomyces cerevisiae. J Cell Biol 98, 934–945. 6365931 10.1083/jcb.98.3.934 PMC2113156

[B4] Aldridge DC, Armstrong JJ, Speake RN, Turner WB (1967). The cytochalasins, a new class of biologically active mould metabolites. Chem Commun 26–27.

[B5] Asadi F, Chakraborty B, Karagiannis J (2017). Latrunculin A-induced perturbation of the actin cytoskeleton mediates Pap1p-dependent induction of the Caf5p efflux pump in *Schizosaccharomyces pombe*. G3 7, 723–730. 28040778 10.1534/g3.116.037903 PMC5295615

[B6] Avasthi P, Onishi M, Karpiak J, Yamamoto R, Mackinder L, Jonikas MC, Sale WS, Shoichet B, Pringle JR, Marshall WF (2014). Actin is required for IFT regulation in *Chlamydomonas reinhardtii*. Curr Biol 24, 2025–2032. 25155506 10.1016/j.cub.2014.07.038 PMC4160380

[B7] Ayscough KR (2000). Endocytosis and the development of cell polarity in yeast require a dynamic F-actin cytoskeleton. Curr Biol 10, 1587–1590. 11137010 10.1016/s0960-9822(00)00859-9

[B8] Ayscough KR, Stryker J, Pokala N, Sanders M, Crews P, Drubin DG (1997). High rates of actin filament turnover in budding yeast and roles for actin in establishment and maintenance of cell polarity revealed using the actin inhibitor latrunculin-A. J Cell Biol 137, 399–416. 9128251 10.1083/jcb.137.2.399 PMC2139767

[B9] Baggett AW, Cournia Z, Han MS, Patargias G, Glass AC, Liu SY, Nolen BJ (2012). Structural characterization and computer-aided optimization of a small-molecule inhibitor of the Arp2/3 complex, a key regulator of the actin cytoskeleton. ChemMedChem 7, 1286–1294. 22623398 10.1002/cmdc.201200104 PMC3531959

[B142] Baell JB (2010). Observations on screening-based research and some concerning trends in the literature. Future Med Chem 2(10), 1529–1546. 21426147 10.4155/fmc.10.237

[B10] Belmont LD, Drubin DG (1998). The yeast V159N actin mutant reveals roles for actin dynamics in vivo. J Cell Biol 142, 1289–1299. 9732289 10.1083/jcb.142.5.1289 PMC2149338

[B11] Belmont LD, Patterson GM, Drubin DG (1999). New actin mutants allow further characterization of the nucleotide-binding cleft and drug-binding sites. J Cell Sci 112, 1325–1336. 10194411 10.1242/jcs.112.9.1325

[B12] Bigge BM, Dougherty LL, Avasthi P (2023a). Lithium-induced ciliary lengthening sparks Arp2/3 complex-dependent endocytosis. Mol Biol Cell 34, ar26. 36753380 10.1091/mbc.E22-06-0219 PMC10092651

[B13] Bigge BM, Rosenthal NE, Avasthi P (2023b). Initial ciliary assembly in *Chlamydomonas* requires Arp2/3 complex-dependent endocytosis. Mol Biol Cell 34, ar24. 36753382 10.1091/mbc.E22-09-0443 PMC10092647

[B14] Bubb MR, Senderowicz AM, Sausville EA, Duncan KL, Korn ED (1994). Jasplakinolide, a cytotoxic natural product, induces actin polymerization and competitively inhibits the binding of phalloidin to F-actin. J Biol Chem 269, 14869–14871. 8195116

[B15] Bubb MR, Spector I, Bershadsky AD, Korn ED (1995). Swinholide A is a microfilament disrupting marine toxin that stabilizes actin dimers and severs actin filaments. J Biol Chem 270, 3463–3466. 7876075 10.1074/jbc.270.8.3463

[B16] Bubb MR, Spector I, Beyer BB, Fosen KM (2000). Effects of jasplakinolide on the kinetics of actin polymerization. An explanation for certain in vivo observations. J Biol Chem 275, 5163–5170. 10671562 10.1074/jbc.275.7.5163

[B17] Cao L, Henty-Ridilla JL, Blanchoin L, Staiger CJ (2016). Profilin-dependent nucleation and assembly of actin filaments controls cell elongation in arabidopsis. Plant Physiol 170, 220–233. 26574597 10.1104/pp.15.01321 PMC4704583

[B18] Cao L, Huang S, Basant A, Mladenov M, Way M (2024). CK-666 and CK-869 differentially inhibit Arp2/3 iso-complexes. EMBO Rep 25, 3221–3239. 39009834 10.1038/s44319-024-00201-x PMC11316031

[B19] Carmely S, Kashman Y (1985). Structure of swinholide-a, a new macrolide from the marine sponge theonella swinhoei. Tetrahedron Lett 26, 511–514.

[B20] Carter SB (1967). Effects of cytochalasins on mammalian cells. Nature 213, 261–264. 6067685 10.1038/213261a0

[B21] Charles-Orszag A, Petek-Seoane NA, Mullins RD (2024). Archaeal actins and the origin of a multi-functional cytoskeleton. J Bacteriol 206, e0034823. 38391233 10.1128/jb.00348-23 PMC10955848

[B22] Cheney KL, White A, Mudianta IW, Winters AE, Quezada M, Capon RJ, Mollo E, Garson MJ (2016). Choose your weaponry: Selective storage of a single toxic compound, latrunculin A, by closely related *Nudibranch Molluscs*. PLoS ONE 11, e0145134. 26788920 10.1371/journal.pone.0145134 PMC4720420

[B23] Clark-Cotton MR, Chen SA, Gomez A, Mulabagal AJ, Perry A, Malhotra V, Onishi M (2025). Imaging-based screen identifies novel natural compounds that perturb cell and chloroplast division in *Chlamydomonas reinhardtii*. Mol Biol Cell 36, br14. 40020179 10.1091/mbc.E24-09-0425 PMC12005104

[B24] Collings DA, Lill AW, Himmelspach R, Wasteneys GO (2006). Hypersensitivity to cytoskeletal antagonists demonstrates microtubule-microfilament cross-talk in the control of root elongation in Arabidopsis thaliana. New Phytol 170, 275–290. 16608453 10.1111/j.1469-8137.2006.01671.x

[B25] Cooper JA (1987). Effects of cytochalasin and phalloidin on actin. J Cell Biol 105, 1473–1478. 3312229 10.1083/jcb.105.4.1473 PMC2114638

[B26] Cossar PJ, Cardoso D, Mathwin D, Russell CC, Chiew B, Hamilton MP, Baker JR, Young KA, Chau N, Robinson PJ, *et al.* (2023). Wiskostatin and other carbazole scaffolds as off target inhibitors of dynamin I GTPase activity and endocytosis. Eur J Med Chem 247, 115001. 36577213 10.1016/j.ejmech.2022.115001

[B27] Craig EW, Mueller DM, Bigge BM, Schaffer M, Engel BD, Avasthi P (2019). The elusive actin cytoskeleton of a green alga expressing both conventional and divergent actins. Mol Biol Cell 30, 2827–2837. 31532705 10.1091/mbc.E19-03-0141 PMC6789165

[B28] Dentler WL, Adams C (1992). Flagellar microtubule dynamics in *Chlamydomonas*: Cytochalasin D induces periods of microtubule shortening and elongation; and colchicine induces disassembly of the distal, but not proximal, half of the flagellum. J Cell Biol 117, 1289–1298. 1607390 10.1083/jcb.117.6.1289 PMC2289510

[B29] Dhanda AS, Vogl AW, Albraiki SE, Otey CA, Beck MR, Guttman JA (2018). Palladin compensates for the Arp2/3 complex and supports actin structures during listeria infections. mBio 9, e02259–17. 29636431 10.1128/mBio.02259-17 PMC5893873

[B30] Dong Y, Shahid-Salles S, Sherling D, Fechheimer N, Iyer N, Wells L, Fechheimer M, Furukawa R (2016). De novo actin polymerization is required for model Hirano body formation in *Dictyostelium*. Biol Open 5, 807–818. 27215322 10.1242/bio.014944 PMC4920178

[B31] Ecke M, Prassler J, Tanribil P, Muller-Taubenberger A, Korber S, Faix J, Gerisch G (2020). Formins specify membrane patterns generated by propagating actin waves. Mol Biol Cell 31, 373–385. 31940262 10.1091/mbc.E19-08-0460 PMC7183788

[B32] Faulstich H, Zobeley S, Heintz D, Drewes G (1993). Probing the phalloidin-binding site of actin. FEBS Lett 318, 218–222. 8440376 10.1016/0014-5793(93)80515-v

[B33] Filipuzzi I, Thomas JR, Pries V, Estoppey D, Salcius M, Studer C, Schirle M, Hoepfner D (2017). Direct interaction of Chivosazole F with actin elicits cell responses similar to latrunculin A but distinct from Chondramide. ACS Chem Biol 12, 2264–2269. 28796488 10.1021/acschembio.7b00385

[B34] Fritz-Laylin LK, Lord SJ, Mullins RD (2017). WASP and SCAR are evolutionarily conserved in actin-filled pseudopod-based motility. J Cell Biol 216, 1673–1688. 28473602 10.1083/jcb.201701074 PMC5461030

[B35] Fujita M, Ichinose S, Kiyono T, Tsurumi T, Omori A (2003). Establishment of latrunculin-A resistance in HeLa cells by expression of R183A D184A mutant beta-actin. Oncogene 22, 627–631. 12555075 10.1038/sj.onc.1206173

[B36] Fujiwara I, Zweifel ME, Courtemanche N, Pollard TD (2018). Latrunculin A accelerates actin filament depolymerization in addition to sequestering actin monomers. Curr Biol 28, 3183–3192.e2. 30270183 10.1016/j.cub.2018.07.082 PMC6179359

[B37] Fukui Y, Katsumaru H (1980). Dynamics of nuclear actin bundle induction by dimethyl sulfoxide and factors affecting its development. J Cell Biol 84, 131–140. 7188610 10.1083/jcb.84.1.131 PMC2110527

[B38] Furstner A, De Souza D, Turet L, Fenster MD, Parra-Rapado L, Wirtz C, Mynott R, Lehmann CW (2007). Total syntheses of the actin-binding macrolides latrunculin A, B, C, M, S and 16-epi-latrunculin B. Chemistry 13, 115–134. 17091520 10.1002/chem.200601135

[B39] Gerisch G, Bretschneider T, Muller-Taubenberger A, Simmeth E, Ecke M, Diez S, Anderson K (2004). Mobile actin clusters and traveling waves in cells recovering from actin depolymerization. Biophys J 87, 3493–3503. 15347592 10.1529/biophysj.104.047589 PMC1304815

[B40] Guerriero CJ, Weisz OA (2007). N-WASP inhibitor wiskostatin nonselectively perturbs membrane transport by decreasing cellular ATP levels. Am J Physiol Cell Physiol 292, C1562–C1566. 17092993 10.1152/ajpcell.00426.2006

[B41] Guha M, Zhou M, Wang YL (2005). Cortical actin turnover during cytokinesis requires myosin II. Curr Biol 15, 732–736. 15854905 10.1016/j.cub.2005.03.042

[B42] Hamaguchi Y, Mabuchi I (1982). Effects of phalloidin microinjection and localization of fluorescein-labeled phalloidin in living sand dollar eggs. Cell Motil 2, 103–113. 7172218 10.1002/cm.970020203

[B43] Haynes EM, Asokan SB, King SJ, Johnson HE, Haugh JM, Bear JE (2015). GMFbeta controls branched actin content and lamellipodial retraction in fibroblasts. J Cell Biol 209, 803–812. 26101216 10.1083/jcb.201501094 PMC4477851

[B143] Heissler SM, Chinthalapudi K, Sellers JR (2015). Kinetic characterization of the sole nonmuscle myosin-2 from the model organism Drosophila melanogaster. The FASEB Journal 29(4), 1456–1466. 25636739 10.1096/fj.14-266742 PMC4396609

[B44] Henson JH, Yeterian M, Weeks RM, Medrano AE, Brown BL, Geist HL, Pais MD, Oldenbourg R, Shuster CB (2015). Arp2/3 complex inhibition radically alters lamellipodial actin architecture, suspended cell shape, and the cell spreading process. Mol Biol Cell 26, 887–900. 25568343 10.1091/mbc.E14-07-1244 PMC4342025

[B45] Hertzer C, Undap N, Papu A, Bhandari D, Aatz S, Kehraus S, Kaligis F, Bara R, Schäberle T, Wägele H, *et al.* (2023). Is a modified actin the key to toxin resistance in the Nudibranch Chromodoris? A biochemical and molecular approach. Diversity 15.

[B46] Hetrick B, Han MS, Helgeson LA, Nolen BJ (2013). Small molecules CK-666 and CK-869 inhibit actin-related protein 2/3 complex by blocking an activating conformational change. Chem Biol 20, 701–712. 23623350 10.1016/j.chembiol.2013.03.019 PMC3684959

[B47] Hirono M, Kumagai Y, Numata O, Watanabe Y (1989). Purification of Tetrahymena actin reveals some unusual properties. Proc Natl Acad Sci USA 86, 75–79. 2521389 10.1073/pnas.86.1.75 PMC286406

[B48] Hoffman JL, Goodenough UW (1980). Experimental dissection of flagellar surface motility in *Chlamydomonas*. J Cell Biol 86, 656–665. 7400220 10.1083/jcb.86.2.656 PMC2111476

[B49] Holthaus L, Lamp D, Gavrisan A, Sharma V, Ziegler AG, Jastroch M, Bonifacio E (2018). CD4(+) T-cell activation, function, and metabolism are inhibited by low concentrations of DMSO. J Immunol Methods 463, 54–60. 30201392 10.1016/j.jim.2018.09.004

[B50] Holzinger A, Meindl U (1997). Jasplakinolide, a novel actin targeting peptide, inhibits cell growth and induces actin filament polymerization in the green alga Micrasterias. Cell Motil Cytoskeleton 38, 365–372. 9415378 10.1002/(SICI)1097-0169(1997)38:4<365::AID-CM6>3.0.CO;2-2

[B51] Hosein RE, Williams SA, Gavin RH (2005). Directed motility of phagosomes in Tetrahymena thermophila requires actin and Myo1p, a novel unconventional myosin. Cell Motil Cytoskeleton 61, 49–60. 15810016 10.1002/cm.20065

[B52] Innocenti M (2023). Investigating mammalian formins with SMIFH2 fifteen years in: Novel targets and unexpected biology. Int J Mol Sci 24, 9058. 37240404 10.3390/ijms24109058 PMC10218792

[B53] Jarett L, Smith RM (1979). Effect of cytochalasin B and D on groups of insulin receptors and on insulin action in rat adipocytes. Possible evidence for a structural relationship of the insulin receptor to the glucose transport system. J Clin Invest 63, 571–579. 438322 10.1172/JCI109338 PMC371990

[B54] Johnston JJ, Wen KK, Keppler-Noreuil K, McKane M, Maiers JL, Greiner A, Sapp JC, Center NIHIS, Demali KA, Rubenstein PA, *et al.* (2013). Functional analysis of a de novo ACTB mutation in a patient with atypical Baraitser-Winter syndrome. Hum Mutat 34, 1242–1249. 23649928 10.1002/humu.22350 PMC3745514

[B55] Kashman Y, Groweiss A, Shmueli U (1980). Latrunculin, a new 2-thiazolidinone macrolide from the marine sponge latrunculia magnifica. Tetrahedron Lett 21, 3629–3632.

[B56] Kato-Minoura T, Hirono M, Kamiya R (1997). Chlamydomonas inner-arm dynein mutant, ida5, has a mutation in an actin-encoding gene. J Cell Biol 137, 649–656. 9151671 10.1083/jcb.137.3.649 PMC2139884

[B57] Kato-Minoura T, Okumura M, Hirono M, Kamiya R (2003). A novel family of unconventional actins in volvocalean algae. J Mol Evol 57, 555–561. 14738314 10.1007/s00239-003-2509-3

[B58] Kelsen A, Kent RS, Snyder AK, Wehri E, Bishop SJ, Stadler RV, Powell C, Martorelli di Genova B, Rompikuntal PK, Boulanger MJ, *et al.* (2023). MyosinA is a druggable target in the widespread protozoan parasite *Toxoplasma gondii*. PLoS Biol 21, e3002110. 37155705 10.1371/journal.pbio.3002110 PMC10185354

[B59] Kepiro M, Varkuti BH, Bodor A, Hegyi G, Drahos L, Kovacs M, Malnasi-Csizmadia A (2012). Azidoblebbistatin, a photoreactive myosin inhibitor. Proc Natl Acad Sci USA 109, 9402–9407. 22647605 10.1073/pnas.1202786109 PMC3386077

[B60] Kepiro M, Varkuti BH, Vegner L, Voros G, Hegyi G, Varga M, Malnasi-Csizmadia A (2014). para-Nitroblebbistatin, the non-cytotoxic and photostable myosin II inhibitor. Angew Chem Int Ed Engl 53, 8211–8215. 24954740 10.1002/anie.201403540

[B61] Klenchin VA, Allingham JS, King R, Tanaka J, Marriott G, Rayment I (2003). Trisoxazole macrolide toxins mimic the binding of actin-capping proteins to actin. Nat Struct Biol 10, 1058–1063. 14578936 10.1038/nsb1006

[B62] Knowles SL, Raja HA, Wright AJ, Lee AML, Caesar LK, Cech NB, Mead ME, Steenwyk JL, Ries LNA, Goldman GH, *et al.* (2019). Mapping the fungal battlefield: Using in situ chemistry and deletion mutants to monitor interspecific chemical interactions between fungi. Front Microbiol 10, 285. 30837981 10.3389/fmicb.2019.00285 PMC6389630

[B63] Kolega J (2004). Phototoxicity and photoinactivation of blebbistatin in UV and visible light. Biochem Biophys Res Commun 320, 1020–1025. 15240150 10.1016/j.bbrc.2004.06.045

[B64] Koonce MP, Euteneuer U, McDonald KL, Menzel D, Schliwa M (1986). Cytoskeletal architecture and motility in a giant freshwater amoeba *Reticulomyxa*. Cell Motil Cytoskeleton 6, 521–533. 3791427 10.1002/cm.970060511

[B65] Kovacs M, Toth J, Hetenyi C, Malnasi-Csizmadia A, Sellers JR (2004). Mechanism of blebbistatin inhibition of myosin II. J Biol Chem 279, 35557–35563. 15205456 10.1074/jbc.M405319200

[B66] Kumari A, Kesarwani S, Javoor MG, Vinothkumar KR, Sirajuddin M (2020). Structural insights into actin filament recognition by commonly used cellular actin markers. EMBO J 39, e104006. 32567727 10.15252/embj.2019104006 PMC7360965

[B67] Li R, Zheng Y, Drubin DG (1995). Regulation of cortical actin cytoskeleton assembly during polarized cell growth in budding yeast. J Cell Biol 128, 599–615. 7860633 10.1083/jcb.128.4.599 PMC2199892

[B68] Lu SE, Novak J, Austin FW, Gu G, Ellis D, Kirk M, Wilson-Stanford S, Tonelli M, Smith L (2009). Occidiofungin, a unique antifungal glycopeptide produced by a strain of Burkholderia contaminans. Biochemistry 48, 8312–8321. 19673482 10.1021/bi900814c PMC2771368

[B69] Lukinavicius G, Reymond L, D'Este E, Masharina A, Gottfert F, Ta H, Guther A, Fournier M, Rizzo S, Waldmann H, *et al.* (2014). Fluorogenic probes for live-cell imaging of the cytoskeleton. Nat Methods 11, 731–733. 24859753 10.1038/nmeth.2972

[B70] MacLean-Fletcher S, Pollard TD (1980). Mechanism of action of cytochalasin B on actin. Cell 20, 329–341. 6893016 10.1016/0092-8674(80)90619-4

[B71] Malla M, Pollard TD, Chen Q (2022). Counting actin in contractile rings reveals novel contributions of cofilin and type II myosins to fission yeast cytokinesis. Mol Biol Cell 33, ar51. 34613787 10.1091/mbc.E21-08-0376 PMC9265160

[B72] Malych R, Folgosa F, Pilatova J, Mikes L, Dohnalek V, Mach J, Matejkova M, Kopecky V, Dolezal P, Sutak R (2025). Eating the brain—A multidisciplinary study provides new insights into the mechanisms underlying the cytopathogenicity of Naegleria fowleri. PLoS Pathog 21, e1012995. 40096149 10.1371/journal.ppat.1012995 PMC11964265

[B73] Maschmann S, Ruban K, Wientapper J, Walter WJ (2020). Phototaxis of the unicellular red alga *Cyanidioschyzon merolae* is mediated by novel actin-driven tentacles. Int J Mol Sci 21, 6209. 32867346 10.3390/ijms21176209 PMC7503314

[B74] Melak M, Plessner M, Grosse R (2017). Actin visualization at a glance. J Cell Sci 130, 525–530. 28082420 10.1242/jcs.189068

[B75] Mendes Pinto I, Rubinstein B, Kucharavy A, Unruh JR, Li R (2012). Actin depolymerization drives actomyosin ring contraction during budding yeast cytokinesis. Dev Cell 22, 1247–1260. 22698284 10.1016/j.devcel.2012.04.015 PMC3376349

[B76] Mikulich A, Kavaliauskiene S, Juzenas P (2012). Blebbistatin, a myosin inhibitor, is phototoxic to human cancer cells under exposure to blue light. Biochim Biophys Acta 1820, 870–877. 22507270 10.1016/j.bbagen.2012.04.003

[B77] Miller HE, Larson CL, Heinzen RA (2018). Actin polymerization in the endosomal pathway, but not on the Coxiella-containing vacuole, is essential for pathogen growth. PLoS Pathog 14, e1007005. 29668757 10.1371/journal.ppat.1007005 PMC5927470

[B78] Mishra M, Kashiwazaki J, Takagi T, Srinivasan R, Huang Y, Balasubramanian MK, Mabuchi I (2013). In vitro contraction of cytokinetic ring depends on myosin II but not on actin dynamics. Nat Cell Biol 15, 853–859. 23770677 10.1038/ncb2781

[B79] Mondal S, Bakthavatsalam D, Steimle P, Gassen B, Rivero F, Noegel AA (2008). Linking Ras to myosin function: RasGEF Q, a Dictyostelium exchange factor for RasB, affects myosin II functions. J Cell Biol 181, 747–760. 18504297 10.1083/jcb.200710111 PMC2396803

[B80] Morton WM, Ayscough KR, McLaughlin PJ (2000). Latrunculin alters the actin-monomer subunit interface to prevent polymerization. Nat Cell Biol 2, 376–378. 10854330 10.1038/35014075

[B81] Moussaoui D, Robblee JP, Robert-Paganin J, Auguin D, Fisher F, Fagnant PM, Macfarlane JE, Schaletzky J, Wehri E, Mueller-Dieckmann C, *et al.* (2023). Mechanism of small-molecule inhibition of *Plasmodium falciparum* myosin A informs antimalarial drug design. Nat Commun 14, 3463. 37308472 10.1038/s41467-023-38976-7 PMC10261046

[B82] Mullins RD, Heuser JA, Pollard TD (1998). The interaction of Arp2/3 complex with actin: Nucleation, high affinity pointed end capping, and formation of branching networks of filaments. Proc Natl Acad Sci USA 95, 6181–6186. 9600938 10.1073/pnas.95.11.6181 PMC27619

[B83] Murthy K, Wadsworth P (2005). Myosin-II-dependent localization and dynamics of F-actin during cytokinesis. Curr Biol 15, 724–731. 15854904 10.1016/j.cub.2005.02.055

[B84] Nair UB, Joel PB, Wan Q, Lowey S, Rould MA, Trybus KM (2008). Crystal structures of monomeric actin bound to cytochalasin D. J Mol Biol 384, 848–864. 18938176 10.1016/j.jmb.2008.09.082 PMC2638586

[B85] Nèeman I, Fishelson L, Kashman Y (1975). Isolation of a new toxin from the sponge Latrunculia magnifica in the Gulf of Aquaba (Red Sea). Marine Biology 30, 293–296.

[B86] Nishimura Y, Shi S, Zhang F, Liu R, Takagi Y, Bershadsky AD, Viasnoff V, Sellers JR (2021). The formin inhibitor SMIFH2 inhibits members of the myosin superfamily. J Cell Sci 134, jcs253708. 33589498 10.1242/jcs.253708 PMC8121067

[B87] Nolen BJ, Tomasevic N, Russell A, Pierce DW, Jia Z, McCormick CD, Hartman J, Sakowicz R, Pollard TD (2009). Characterization of two classes of small molecule inhibitors of Arp2/3 complex. Nature 460, 1031–1034. 19648907 10.1038/nature08231 PMC2780427

[B88] Onishi M, Pecani K, Jones Tt, Pringle JR, Cross FR (2018). F-actin homeostasis through transcriptional regulation and proteasome-mediated proteolysis. Proc Natl Acad Sci USA 115, E6487–E6496. 29941587 10.1073/pnas.1721935115 PMC6048543

[B89] Onishi M, Pringle JR, Cross FR (2016). Evidence that an unconventional actin can provide essential F-actin function and that a surveillance system monitors F-actin integrity in *Chlamydomonas*. Genetics 202, 977–996. 26715672 10.1534/genetics.115.184663 PMC4788133

[B90] Onishi M, Umen JG, Cross FR, Pringle JR (2020). Cleavage-furrow formation without F-actin in *Chlamydomonas*. Proc Natl Acad Sci USA 117, 18511–18520. 32690698 10.1073/pnas.1920337117 PMC7414186

[B91] Paredez AR, Assaf ZJ, Sept D, Timofejeva L, Dawson SC, Wang CJ, Cande WZ (2011). An actin cytoskeleton with evolutionarily conserved functions in the absence of canonical actin-binding proteins. Proc Natl Acad Sci USA 108, 6151–6156. 21444821 10.1073/pnas.1018593108 PMC3076823

[B92] Peterson JR, Bickford LC, Morgan D, Kim AS, Ouerfelli O, Kirschner MW, Rosen MK (2004). Chemical inhibition of N-WASP by stabilization of a native autoinhibited conformation. Nat Struct Mol Biol 11, 747–755. 15235593 10.1038/nsmb796

[B93] Pfanzelter J, Mostowy S, Way M (2018). Septins suppress the release of vaccinia virus from infected cells. J Cell Biol 217, 2911–2929. 29921601 10.1083/jcb.201708091 PMC6080921

[B94] Pospich S, Kumpula EP, von der Ecken J, Vahokoski J, Kursula I, Raunser S (2017). Near-atomic structure of jasplakinolide-stabilized malaria parasite F-actin reveals the structural basis of filament instability. Proc Natl Acad Sci USA 114, 10636–10641. 28923924 10.1073/pnas.1707506114 PMC5635891

[B95] Pospich S, Merino F, Raunser S (2020). Structural effects and functional implications of Phalloidin and Jasplakinolide binding to actin filaments. Structure 28, 437–449.e5. 32084355 10.1016/j.str.2020.01.014

[B96] Poulsen NC, Spector I, Spurck TP, Schultz TF, Wetherbee R (1999). Diatom gliding is the result of an actin-myosin motility system. Cell Motil Cytoskeleton 44, 23–33. 10470016 10.1002/(SICI)1097-0169(199909)44:1<23::AID-CM2>3.0.CO;2-D

[B97] Pourdanandeh M, Sechet V, Carpentier L, Reveillon D, Herve F, Hubert C, Hess P, Selander E (2025). Effects of copepod chemical cues on intra- and extracellular toxins in two species of *Dinophysis*. Harmful Algae 142, 102793. 39947851 10.1016/j.hal.2024.102793

[B98] Procaccio V, Salazar G, Ono S, Styers ML, Gearing M, Davila A, Jimenez R, Juncos J, Gutekunst CA, Meroni G, *et al.* (2006). A mutation of beta -actin that alters depolymerization dynamics is associated with autosomal dominant developmental malformations, deafness, and dystonia. Am J Hum Genet 78, 947–960. 16685646 10.1086/504271 PMC1474101

[B99] Proksch P (1994). Defensive roles for secondary metabolites from marine sponges and sponge-feeding nudibranchs. Toxicon 32, 639–655. 7940572 10.1016/0041-0101(94)90334-4

[B100] Prostak SM, Robinson KA, Titus MA, Fritz-Laylin LK (2021). The actin networks of chytrid fungi reveal evolutionary loss of cytoskeletal complexity in the fungal kingdom. Curr Biol 31, 1192–1205.e6. 33561386 10.1016/j.cub.2021.01.001 PMC8812817

[B101] Ravichandran A, Geng M, Hull KG, Li J, Romo D, Lu SE, Albee A, Nutter C, Gordon DM, Ghannoum MA, *et al.* (2019). A novel actin-binding drug with in vivo efficacy. Antimicrob Agents Chemother 63, e01585–18. 30323040 10.1128/AAC.01585-18 PMC6325233

[B102] Riviere JB, van Bon BW, Hoischen A, Kholmanskikh SS, O'Roak BJ, Gilissen C, Gijsen S, Sullivan CT, Christian SL, Abdul-Rahman OA, *et al.* (2012). De novo mutations in the actin genes ACTB and ACTG1 cause Baraitser–Winter syndrome. Nat Genet 44, 440–444, S1–S2. 22366783 10.1038/ng.1091 PMC3677859

[B103] Rizvi SA, Neidt EM, Cui J, Feiger Z, Skau CT, Gardel ML, Kozmin SA, Kovar DR (2009). Identification and characterization of a small-molecule inhibitor of formin-mediated actin assembly. Chem Biol 16, 1158–1168. 19942139 10.1016/j.chembiol.2009.10.006 PMC2784894

[B104] Robinson KA, Prostak SM, Campbell Grant EH, Fritz-Laylin LK (2022). Amphibian mucus triggers a developmental transition in the frog-killing chytrid fungus. Curr Biol 32, 2765–2771.e4. 35472310 10.1016/j.cub.2022.04.006

[B105] Rotty JD, Brighton HE, Craig SL, Asokan SB, Cheng N, Ting JP, Bear JE (2017). Arp2/3 complex is required for macrophage integrin functions but is dispensable for FcR Phagocytosis and in vivo motility. Dev Cell 42, 498–513.e6. 28867487 10.1016/j.devcel.2017.08.003 PMC5601320

[B106] Saiwai K, Okuno T, Fujioka H, Furuya M (1983). The relation between the phytotoxicity of cytochalasin E and its molecular structure. Japanese J Phytopathol 49, 262–265.

[B107] Sakamoto T, Limouze J, Combs CA, Straight AF, Sellers JR (2005). Blebbistatin, a myosin II inhibitor, is photoinactivated by blue light. Biochemistry 44, 584–588. 15641783 10.1021/bi0483357

[B108] Sawitzky H, Liebe S, Willingale-Theune J, Menzel D (1999). The antiproliferative agent jasplakinolide rearranges the actin cytoskeleton of plant cells. Eur J Cell Biol 78, 424–433. 10430024 10.1016/S0171-9335(99)80085-5

[B109] Schmit AC, Lambert AM (1990). Microinjected fluorescent phalloidin in vivo reveals the F-actin dynamics and assembly in higher plant mitotic cells. Plant Cell 2, 129–138. 2136631 10.1105/tpc.2.2.129 PMC159870

[B110] Schroeder TE (1970). The contractile ring. I. Fine structure of dividing mammalian (HeLa) cells and the effects of cytochalasin B. Z Zellforsch Mikrosk Anat 109, 431–449. 5498229

[B111] Schwarzerova K, Vondrakova Z, Fischer L, Borikova P, Bellinvia E, Eliasova K, Havelkova L, Fiserova J, Vagner M, Opatrny Z (2010). The role of actin isoforms in somatic embryogenesis in Norway spruce. BMC Plant Biol 10, 89. 20478025 10.1186/1471-2229-10-89 PMC3095357

[B112] Sehring IM, Mansfeld J, Reiner C, Wagner E, Plattner H, Kissmehl R (2007a). The actin multigene family of *Paramecium tetraurelia*. BMC Genomics 8, 82. 17391512 10.1186/1471-2164-8-82 PMC1852557

[B113] Sehring IM, Reiner C, Mansfeld J, Plattner H, Kissmehl R (2007b). A broad spectrum of actin paralogs in *Paramecium tetraurelia* cells display differential localization and function. J Cell Sci 120, 177–190. 17164292 10.1242/jcs.03313

[B114] Sharma GM, Michaels L, Burkholder PR (1968). Goniodomin, a new antibiotic from a dinoflagellate. J Antibiot 21, 659–664. 10.7164/antibiotics.21.659 5716842

[B115] Sheahan KL, Cordero CL, Satchell KJ (2004). Identification of a domain within the multifunctional Vibrio cholerae RTX toxin that covalently cross-links actin. Proc Natl Acad Sci USA 101, 9798–9803. 15199181 10.1073/pnas.0401104101 PMC470754

[B116] Shu S, Liu X, Korn ED (2005). Blebbistatin and blebbistatin-inactivated myosin II inhibit myosin II-independent processes in *Dictyostelium*. Proc Natl Acad Sci USA 102, 1472–1477. 15671182 10.1073/pnas.0409528102 PMC547870

[B117] Spector I, Shochet NR, Kashman Y, Groweiss A (1983). Latrunculins: Novel marine toxins that disrupt microfilament organization in cultured cells. Science 219, 493–495. 6681676 10.1126/science.6681676

[B144] Spector I, Shochet NR, Blasberger D, Kashman Y (1989). Latrunculins–novel marine macrolides that disrupt microfilament organization and affect cell growth: I. Comparison with cytochalasin D. Cell Motil Cytoskeleton 13(3), 127–144. 2776221 10.1002/cm.970130302

[B118] Straight AF, Cheung A, Limouze J, Chen I, Westwood NJ, Sellers JR, Mitchison TJ (2003). Dissecting temporal and spatial control of cytokinesis with a myosin II Inhibitor. Science 299, 1743–1747. 12637748 10.1126/science.1081412

[B119] Sun SC, Wang ZB, Xu YN, Lee SE, Cui XS, Kim NH (2011). Arp2/3 complex regulates asymmetric division and cytokinesis in mouse oocytes. PLoS ONE 6, e18392. 21494665 10.1371/journal.pone.0018392 PMC3072972

[B120] Suzuki K, Kawazu T, Mita T, Takahashi H, Itoh R, Toda K, Kuroiwa T (1995). Cytokinesis by a contractile ring in the primitive red alga *Cyanidium caldarium* RK-1. Eur J Cell Biol 67, 170–178. 7664758

[B121] Toshima JY, Furuya E, Nagano M, Kanno C, Sakamoto Y, Ebihara M, Siekhaus DE, Toshima J (2016). Yeast Eps15-like endocytic protein Pan1p regulates the interaction between endocytic vesicles, endosomes and the actin cytoskeleton. Elife 5. 10.7554/eLife.10276 PMC4775215 26914139

[B122] Tsurushima T, Don LD, Kawashima K, Murakami J, Nakayashiki H, Tosa Y, Mayama S (2005). Pyrichalasin H production and pathogenicity of Digitaria-specific isolates of Pyricularia grisea. Mol Plant Pathol 6, 605–613. 20565683 10.1111/j.1364-3703.2005.00309.x

[B123] Varkuti BH, Kepiro M, Horvath IA, Vegner L, Rati S, Zsigmond A, Hegyi G, Lenkei Z, Varga M, Malnasi-Csizmadia A (2016). A highly soluble, non-phototoxic, nonfluorescent blebbistatin derivative. Sci Rep 6, 26141. 27241904 10.1038/srep26141 PMC4886532

[B124] Velle KB, Campellone KG (2018). Enteropathogenic E. coli relies on collaboration between the formin mDia1 and the Arp2/3 complex for actin pedestal biogenesis and maintenance. PLoS Pathog 14, e1007485. 30550556 10.1371/journal.ppat.1007485 PMC6310289

[B125] Velle KB, Fritz-Laylin LK (2020). Conserved actin machinery drives microtubule-independent motility and phagocytosis in Naegleria. J Cell Biol 219, e202007158. 32960946 10.1083/jcb.202007158 PMC7594500

[B126] Velle KB, Swafford AJM, Garner E, Fritz-Laylin LK (2024). Actin network evolution as a key driver of eukaryotic diversification. J Cell Sci 137, jcs261660. 39120594 10.1242/jcs.261660 PMC12050087

[B127] Verheijen M, Lienhard M, Schrooders Y, Clayton O, Nudischer R, Boerno S, Timmermann B, Selevsek N, Schlapbach R, Gmuender H, *et al.* (2019). DMSO induces drastic changes in human cellular processes and epigenetic landscape in vitro. Sci Rep 9, 4641. 30874586 10.1038/s41598-019-40660-0 PMC6420634

[B128] Wagner G, Haupt W, Laux A (1972). Reversible inhibition of chloroplast movement by cytochalasin B in the green alga mougeofia. Science 176, 808–809. 17795409 10.1126/science.176.4036.808

[B129] Wang S, Crevenna AH, Ugur I, Marion A, Antes I, Kazmaier U, Hoyer M, Lamb DC, Gegenfurtner F, Kliesmete Z, *et al.* (2019). Actin stabilizing compounds show specific biological effects due to their binding mode. Sci Rep 9, 9731. 31278311 10.1038/s41598-019-46282-w PMC6611809

[B130] Wehland J, Osborn M, Weber K (1977). Phalloidin-induced actin polymerization in the cytoplasm of cultured cells interferes with cell locomotion and growth. Proc Natl Acad Sci USA 74, 5613–5617. 341163 10.1073/pnas.74.12.5613 PMC431831

[B131] Wessells NK, Spooner BS, Ash JF, Bradley MO, Luduena MA, Taylor EL, Wrenn JT, Yamada K (1971). Microfilaments in cellular and developmental processes. Science 171, 135–143. 5538822 10.1126/science.171.3967.135

[B132] Wieland T (1968). Poisonous principles of mushrooms of the genus Amanita. Four-carbon amines acting on the central nervous system and cell-destroying cyclic peptides are produced. Science 159, 946–952. 4865716 10.1126/science.159.3818.946

[B133] Wilson SM, Pickett-Heaps JD, West JA (2002). Fertilization and the cytoskeleton in the red algaBostrychia moritziana(Rhodomelaceae, Rhodophyta). Eur J Phycol 37, 509–522.

[B134] Wirshing ACE, Colarusso AV, Lew DJ (2025). A genetic strategy to allow detection of F-actin by phalloidin staining in diverse fungi. bioRxiv. 10.1128/msphere.00517-25 PMC12570480 41020595

[B135] Wulf E, Deboben A, Bautz FA, Faulstich H, Wieland T (1979). Fluorescent phallotoxin, a tool for the visualization of cellular actin. Proc Natl Acad Sci USA 76, 4498–4502. 291981 10.1073/pnas.76.9.4498 PMC411604

[B136] Xu L, Cao L, Li J, Staiger CJ (2024). Cooperative actin filament nucleation by the Arp2/3 complex and formins maintains the homeostatic cortical array in Arabidopsis epidermal cells. Plant Cell 36, 764–789. 38057163 10.1093/plcell/koad301 PMC10896301

[B137] Yasumoto T, Murata M, Oshima Y, Matsumoto GK, Clardy J (1984). Diarrhetic shellfish poisoning. Seafood Toxins. 207–214. Washington, DC: ACS Symposium Series; American Chemical Society.

[B138] Yi K, Unruh JR, Deng M, Slaughter BD, Rubinstein B, Li R (2011). Dynamic maintenance of asymmetric meiotic spindle position through Arp2/3-complex-driven cytoplasmic streaming in mouse oocytes. Nat Cell Biol 13, 1252–1258. 21874009 10.1038/ncb2320 PMC3523671

[B139] Yumura S, Hashima S, Muranaka S (2014). Myosin II does not contribute to wound repair in *Dictyostelium* cells. Biol Open 3, 966–973. 25238760 10.1242/bio.20149712 PMC4197445

[B140] Zackroff RV, Hufnagel LA (2002). Induction of anti-actin drug resistance in Tetrahymena. J Eukaryot Microbiol 49, 475–477. 12503683 10.1111/j.1550-7408.2002.tb00231.x

[B141] Zigmond SH, Hirsch JG (1972). Cytochalasin B: Inhibition of D-2-deoxyglucose transport into leukocytes and fibroblasts. Science 176, 1432–1434. 5033651 10.1126/science.176.4042.1432

